# Exploring the evolution of multidrug resistance patterns in ESKAPEE pathogens using association mining: Key to antibiotic stewardship?

**DOI:** 10.12688/wellcomeopenres.24103.2

**Published:** 2026-02-20

**Authors:** Sasikala Rajendran, Srimathy Ramachandran, Akilandeswari Ramu, Yashwenth S, Surya Murugan MS, Vigneshwar Ramakrishnan, Suma Mohan S

**Affiliations:** 1 DBT-Bioinformatics Center, School of Chemical & Biotechnology, SASTRA Deemed to be University, Shanmugha Arts Science Technology and Research Academy, Thanjavur, Tamil Nadu, 613401, India; 2 Computational Biology lab, School of Chemical and Biotechnology,, Shanmugha Arts Science Technology and Research Academy, Thanjavur, Tamil Nadu, 613401, India; 3 Computational Molecular Biophysics Laboratory, School of Chemical & Biotechnology, Shanmugha Arts Science Technology and Research Academy, Thanjavur, Tamil Nadu, 613401, India; 4 School of Computing, Shanmugha Arts Science Technology and Research Academy, Thanjavur, Tamil Nadu, 613401, India; 5 School of Electrical & Electronics Engineering,, Shanmugha Arts Science Technology and Research Academy, Thanjavur, Tamil Nadu, 613401, India

**Keywords:** MERIT, AMR, ESKAPEE, shared MDR, Vivli AMR Data Challenge

## Abstract

**Background:**

Shared Multidrug resistance (sMDR) is a major challenge in antimicrobial therapy, particularly among ESKAPEE pathogens, as these bacteria develop resistance to multiple antimicrobial agents via mutations, recombination, or gene transfer. These antimicrobial resistance (AMR) mechanisms evolve and require constant surveillance. Understanding sMDR patterns in large antimicrobial surveillance datasets is complex because of the multifaceted nature of the data.

**Methods:**

We explored Pfizer-Atlas (2004 – 2022) and Venatorx-GEARS (2018-2022) datasets from the Vivli platform, focusing on ESKAPEE pathogens. Descriptive data analysis, time-trend analysis, and association rule generation through the Apriori algorithm were performed using Python 3.10 libraries pandas, matplotlib, seaborn, scipy.stats, and mlxtend libraries. The best rule set that explored sMDR patterns was visualized in a network format using NetworkX: 3.2 package.

**Results:**

“MERIT- Multidrug ESKAPEE Resistance Insights and Tracker” dashboard was created for user-friendly visualization of antimicrobial surveillance datasets, trends in antimicrobial susceptibility profile (ASP) over years, interactive widgets to see the ASP by country and by pathogens, and Network to see significant rules as a result of association rule mining in categories by age, year and country.

**Conclusion:**

Time trend analysis revealed a decline in meropenem and piperacillin-tazobactam resistance to *Enterococcus faecium* and doripenem for *Pseudomonas aeruginosa*, while resistance to imipenem ( *Klebsiella pneumoniae*, *Acinetobacter baumannii*, and *Enterobacter* species) and meropenem ( *Staphylococcus aureus*, *A. baumannii*, and *Enterobacter* species) increased. Association rule mining identified sMDR patterns, such as meropenem and levofloxacin resistance, in *S. aureus*, *K. pneumoniae*, *P. aeruginosa* and *A. baumannii.* Thus, our findings from the data challenge could aid healthcare professionals in making informed decisions regarding antibiotic use.

## Plain Language summary

The emergence of antimicrobial resistance among ESKAPEE pathogens ( *Enterococcus faecium, Staphylococcus aureus, Klebsiella pneumoniae, Acinetobacter baumannii, Pseudomonas aeruginosa, Enterobacter species* and *Escherichia coli*) poses a severe threat to global health. This study aimed to identify shared multidrug-resistant (sMDR) patterns in the antimicrobial surveillance datasets of Pfizer-ATLAS and Venatorx-GEARS available on the Vivli AMR Data challenge platform. Understanding sMDR is crucial for understanding resistance mechanisms, optimizing antibiotic stewardship, and developing targeted interventions to combat AMR. Descriptive data analytics to examine antibiotic resistance profiles across age groups, geographical regions, and ESKAPEE pathogens provided key insights into resistance patterns. The MERIT dashboard ( https://merit.sastra.edu/) was developed to offer a powerful network-based model to visualize and track the prevalence of specific antibiotic resistance profiles of ESKAPEE pathogens, providing actionable insights into evolving resistance patterns. Together, these findings can facilitate end users, including clinicians, researchers, public health professionals, and policymakers, to visualize and interpret the trends for appropriate actions.

## Introduction

Antimicrobial Resistance (AMR) is a global challenge, contributing to approximately 4.71 million deaths in 2021 globally ( Naghavi et al., 2024). The World Health Organization (WHO) estimated 1 to 3.4 trillion (US$) in annual losses to gross domestic product (GDP) by 2030 and an additional 1 trillion (US$) in healthcare costs by 2050 ( WHO, 2024) because of AMR. Further, AMR disproportionately affects low- and middle-income countries compared with high-income nations. Among the AMR pathogens, the ESKAPEE group ( *Enterococcus faecium, Staphylococcus aureus, Klebsiella pneumoniae, Acinetobacter baumannii, Pseudomonas aeruginosa*, *Enterobacter* species, and *Escherichia coli*) is of particular concern because of its ability to evade standard antibiotic treatments. These pathogens exhibit resistance that arises from diverse natural and genetic processes, including natural resistance, acquired resistance, co-resistance ( Chapman, 2003; Baker-Austin et al., 2006), multidrug resistance (MDR), and pan-drug resistance ( Magiorakos et al., 2012). Resistance mechanisms include reduced drug uptake, target site alterations, and the action of efflux pumps ( Munita et al., 2016). MDR refers to the ability of a species to resist various classes of antibiotics. Shared MDR refers to the sharing of similar patterns of resistance mechanisms in multiple species against different classes of antibiotics, as a result of horizontal gene transfer. Co-resistance refers to the mechanism of resistance when a single organism is resistant to multiple drugs, often due to the presence of multiple resistance genes that are located together on the same genetic element. Identifying these resistance patterns requires comprehensive datasets that capture the prevalence and trends of antibiotic resistance in diverse settings. Vivli, a nonprofit organization, provides a platform for sharing AMR surveillance data and facilitating cross-regional comparisons and collaborative research. However, unearthing MDR patterns is a challenging task because of the multifaceted nature of the data, which is influenced by biological complexity, data variability due to geographical location, various strains, and dynamic interactions between resistance mechanisms, human behavior, and environmental factors ( Prestinaci, Pezzotti and Pantosti, 2015). To address this challenge, an interdisciplinary approach integrating robust data collection and advanced computational models is crucial for addressing the evolving nature of AMR. Association rule mining (ARM) is a powerful tool for uncovering significant relationships within extensive datasets. Unlike traditional analytical methods, this approach simplifies complex data by focusing on the most prevalent and relevant relationships, enabling the efficient analysis of MDR patterns. By identifying hidden patterns and high-risk antibiotic combinations, association rule mining offers a data-driven framework for understanding shared MDR patterns in antimicrobial surveillance data. Recent studies that utilized association rule (AR) mining to understand the complex resistance patterns in AMR have been widely employed, aiding in the development of antimicrobial stewardship strategies. For example, a study that analyzed data collected from a hospital in New York between 2008 and 2018 using association rule mining revealed a frequent shared occurrence of clindamycin and tetracycline resistance in skin- and skin-related infections ( Cazer et al., 2021). In another study, AMR profiles of patients with stone disease were explored using AR and helped to identify antibiotic resistance patterns that play a crucial role in managing UTI in these patients ( Manolitsis et al., 2022).

Previously, the Vivli AMR platform was extensively used to identify growing resistance in pediatric AMR patterns, especially among gram-negative pathogens. Variations in minimum inhibitory concentration (MIC) distribution have been highlighted across various patient groups ( Wildfire et al., 2024). It has also provided an AI-driven web-based decision support system to predict antibacterial and antifungal susceptibility ( Mutisya and Kanguha, 2024). It has also been used to examine AMR trends in broad-spectrum antibiotics against *Enterobacteriaceae* in India. It presents an algorithm, PATHFINDER, that assists healthcare in decision-making by predicting resistance genes through interactive dashboards ( Agboeze et al., 2024).

The present study aimed to develop a user-friendly dashboard for uncovering hidden patterns shared MDR and to identify specific antibiotic combinations that are prone to resistance across ESKAPEE pathogens. By leveraging association rule mining, this study sought to investigate how resistance patterns evolve over time and vary across regions, forecasting potential future resistance scenarios. Additionally, it aims to track trends in antibiotic resistance among ESKAPEE pathogens over time, across different age groups, and geographical locations. By examining regional variations in antimicrobial resistance patterns, this study provides critical insights for forecasting future trends and laying a foundation for effective, evidence-based public health interventions.

## Methods

**Figure 1.**
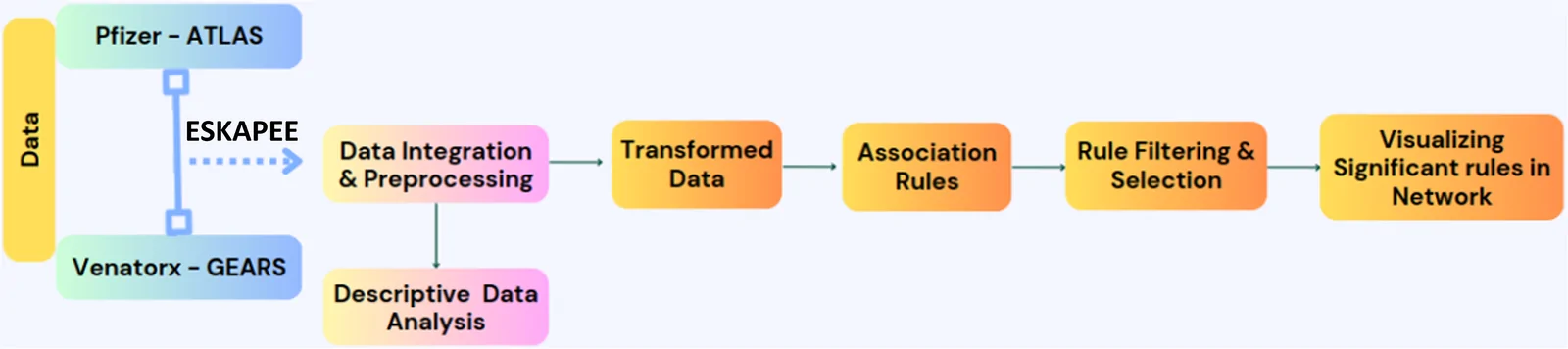
Schematic workflow of the data challenge for exploration of MDR patterns among ESKAPEE pathogens.

To develop a user-friendly dashboard that facilitates the identification of shared antibiotic resistance patterns, and highlights specific antibiotic combinations that are more prone to resistance across ESKAPEE pathogens. The dashboard also enables users to track trends in antimicrobial susceptibility profiles (ASP) over the years. Interactive widgets are given to explore ASP by country and pathogen, making it easier to navigate the dashboard. This interactive visualization supports effective data interpretation and informed decision-making in antimicrobial resistance analysis. To achieve this, we performed the workflow illustrated in Figure 1.

### Data collection

Data for the current study were retrieved from two AMR surveillance programs: ATLAS (Antimicrobial Testing Leadership and Surveillance) (2004 – 2022) and GEARS (Global Evaluation of Antimicrobial Resistance via Surveillance) program (2018-2022) through the Vivli Center for Global Clinical Research Data ( https://amr.vivli.org). The ATLAS program, conducted by Pfizer across 83 countries, contains 917,049 antibiotic isolates and monitors changes in antibiotic susceptibility, bacterial resistance trends, and the emergence of new resistance mechanisms for both marketed and in-development antibiotics. The GEARS program conducted by Venatorx across 60 countries contains 29,365 isolates from community- and hospital-associated infection sources, distributed globally, with a focus on the United States and Europe. In both programs, samples were obtained from patients across various hospital departments, including General Medicine, Medical Intensive Care Unit (ICU), Surgical ICU, Pediatric ICU, General ICU. The isolates were sourced from diverse infection sites, including respiratory tract infections, urinary tract infections, bloodstream infections, intra-abdominal infections, and skin and soft tissue infections. The organisms included were a broad range of Gram-negative and Gram-positive pathogens; however, we took isolates with respect to ESKAPEE pathogens.

### Dataset cleaning and wrangling

The two datasets, Pfizer-ATLAS (2004–2022) and Venatorx-GEARS (2018–2022), were integrated for analysis based on the availability of antibiotic details. Isolates lacking minimum inhibitory concentration (MIC) values or MIC phenotype details (e.g., Resistant or Susceptible) were excluded. For antibiotics with MIC values but missing phenotype information, susceptibility phenotypes were manually assigned using MIC breakpoints from the Clinical and Laboratory Standards Institute (CLSI) guidelines for the corresponding year. The MIC phenotype data were further standardized into two categories: “S” (Susceptible) and “R” (Intermediate and Resistant). Sample sources with less than 1% frequency, such as skeletal tissue, ear, eye, nose, vomit, throat, lymphatic fluid, muscle, kidney, colon, mouth, heart, nails, brain, ovary, placenta, testis, and fallopian tubes were grouped under the “Other” category. Additionally, patient ages were categorized into predefined age groups (0–2, 3–12, 13–18, 19–64, 65–84, and 85+ years) to facilitate age-based analyses.

### Descriptive data analysis

Frequency and percentages were used to describe the sociodemographic variables, including overall trends, yearly distributions, hospital unit-specific data, and infection-site-related data. Frequency and percentage differences between variables and combo charts were generated using Plotly (v5.12.0) ( Plotly Technologies Inc. (2015)) for effective visualization. Descriptive data analysis was performed using the Pandas library ( McKinney et al., 2010), which is widely used for data manipulation, and visualizations were created using Matplotlib (v3.7.1) ( Hunter, 2007) and Seaborn (v0.12.2) ( Waskom, 2021). To analyze trends over time, chi-square trend analysis was performed using the scipy.stats library ( Virtanen et al., 2020) (v1.14.1) to determine whether there was a statistically significant change in the percentage of resistance over the period 2004–2022. Statistical significance was set at P <0.05.

### Association rule mining

In this study, each isolate was treated as a transaction and each antimicrobial was treated as an item. Resistance to an antimicrobial was denoted with the suffix “_R,” susceptibility with “_S,” and these were encoded as “1,” while missing data was encoded as “0.” An itemset represented a combination of resistance to different antimicrobials, and a transaction contained an itemset if all items within it were present in the transaction. The support count of an item set refers to the number of transactions containing that item set, while support is defined as the proportion of such transactions (e.g., a support count of 3/8 corresponds to a support value of 0.3). Frequent itemsets were identified as combinations of resistances occurring together in a significant number of transactions, with a support threshold of 0.3 across years, age groups, and geographical locations. Association rules were generated from the frequent item sets using the Apriori algorithm ( Agrawal et al., 1993) implemented via the mlxtend (v0.19.0) library ( Raschka, 2018) in Python. Rules with lower confidence values were excluded. Frequent itemsets were further partitioned into two non-overlapping subsets: antecedents and consequences. Rules containing at least two antimicrobials were extracted from the dataset and filtered using quality metrics, including support (≥0.3), lift (≥2), confidence (≥0.80), and Zhang’s metric (>90) to derive the best rules. When the default condition did not generate sufficient rules a lower support threshold (>0.1) was used to identify both ‘Resistant–Resistant’ and ‘Resistant–Susceptible’ patterns. These rules were utilized to identify patterns of antimicrobial resistance across years, age groups, and geographical locations. The final best rule set was decomposed into antimicrobial nodes and undirected edges, with the rules represented as weighted edges using the NetworkX (v3.2) package ( Hagberg et al., 2008).

### Dashboard Creation

Streamlit is a free and open-source framework for building machine-learning and data-science web applications. It is compatible with Python libraries, such as pandas, matplotlib, seaborn, plotly, and Keras. We used the Streamlit framework (1.43.2) to visualize the exploratory data analysis (EDA) and associated the mining results on the dashboard. The message “No rules extracted” is displayed when the data supporting a query are insufficient or fail to meet the predefined threshold criteria.

## Results

### MERIT dashboard overview

**Figure 2.**
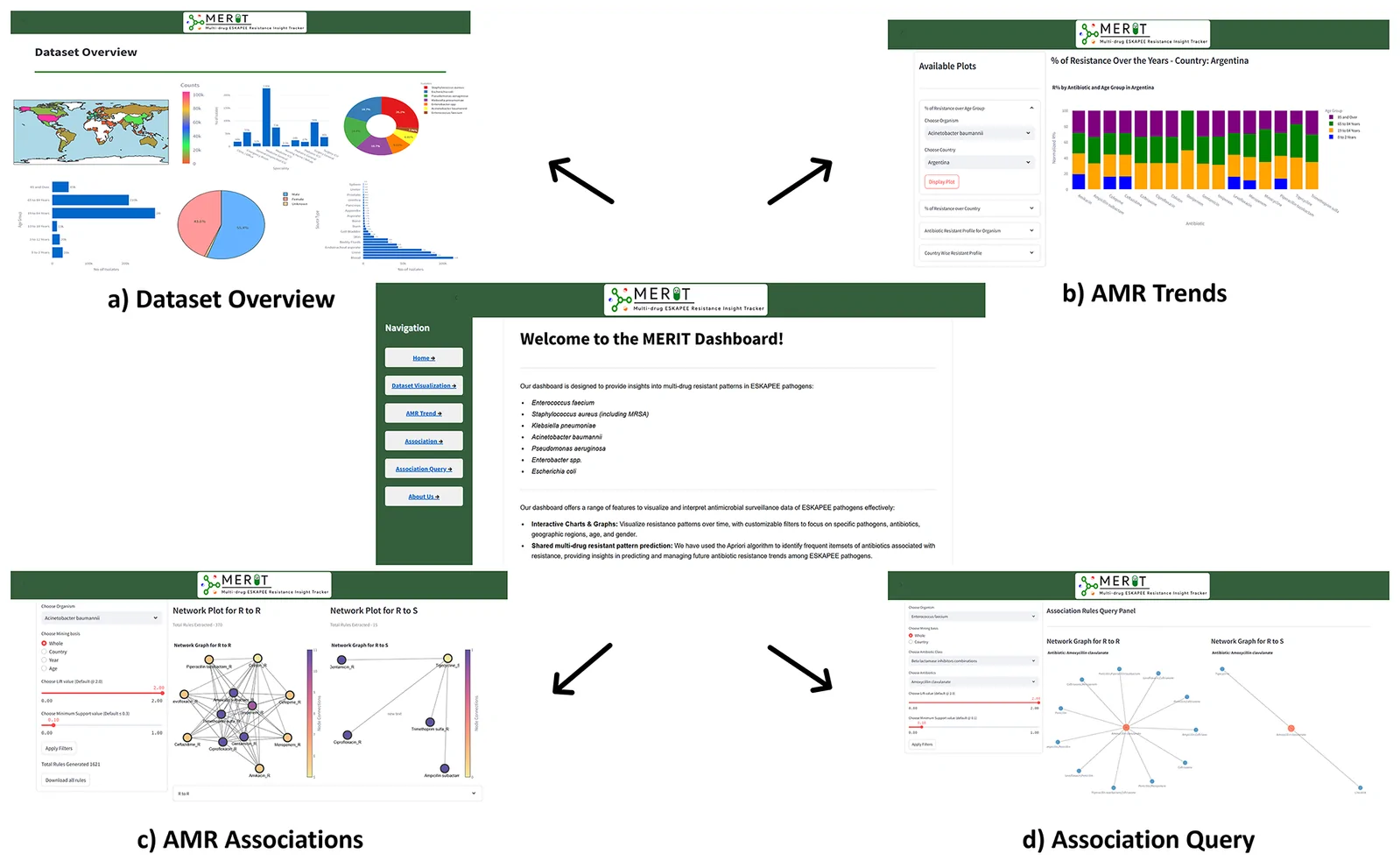
MERIT dashboard features a. Dataset overview b. AMR Trends c. AMR Associations d. Association Query.

The MERIT dashboard aims to provide a shared multidrug resistance pattern. Our dashboard consists of four pages to visualize and interpret the antimicrobial surveillance data of ESKAPEE pathogens ( Figure 2). The “Dataset overview” visualization tab provides an overview of the data used in this study. The “AMR trends” tab provides a resistance profile with age group, country, organism, and country. The “AMR Associations” tab provides shared resistance within the selected species based on age, year, and country. The “Association query” tab provides customization options to allow the user to select the organism and the interested antibiotics to see the resistance and susceptibility pattern.

**Figure 3.**
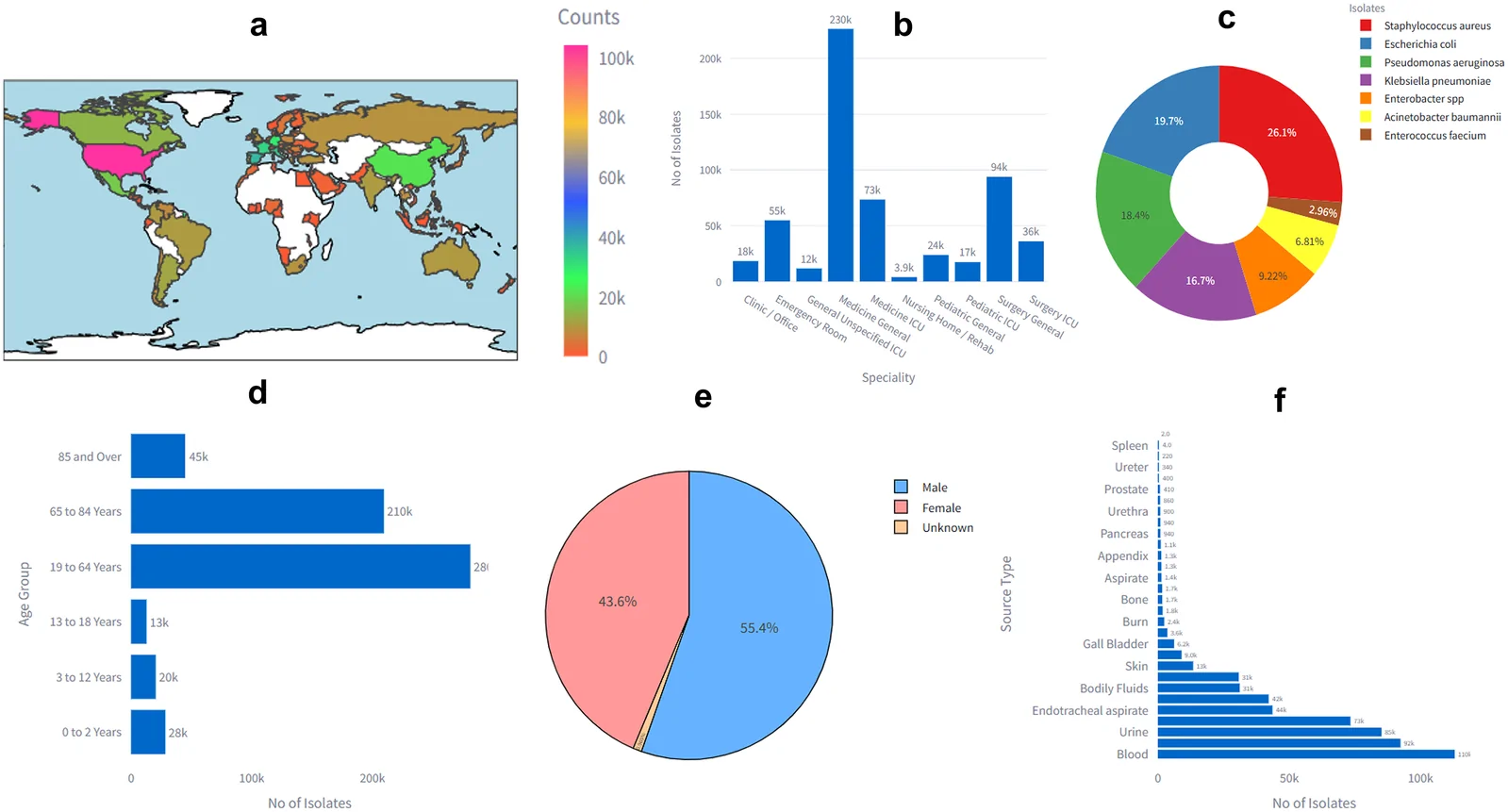
Integrated dataset distribution overview of ESKAPEE pathogens: a. Geographical, b. Specialty, c. Organism, d. Age group, e. Gender, f. Source type

The integrated Pfizer-Atlas and Venatorx-GEARS dataset comprised 605,118 isolates for the seven major ESKAPEE pathogens: *S. aureus* (26%), *E. coli* (20%), *P. aeruginosa* (18%), *K. pneumonia* (17%), *Enterobacter* species (9%), *A. baumannii* (7%), and *E. faecium* (3%) for the years 2004–2022 from 83 countries. Among these, *S. aureus* accounted for the greatest number of isolates in the datasets, followed by *E. coli* ( Figure 3a). Samples were collected from 10 different specialty wards in which Medicine General contributed the most isolates (40.86%), followed by Surgery General (16.70%) and Medicine ICU (12.97%) ( Figure 3b). Further samples were obtained from 36 different sources, of which the majority were collected from blood (18.6%), wounds (15.2%), and urine (14%) ( Figure 3c). In terms of age-wise distribution, 46% of the isolates were from individuals in the 19–64 years age group, followed by 35% from the 65 to 84 years age group ( Figure 3d). In the gender-wise distribution, 43.6% of the isolates were from females and 55.4% were from the male ( Figure 3e). Among the countries, the United States contributed the highest proportion of isolates (17%), followed by Spain (6%), Italy (5.4%), France (5.4%), and Germany (4.8%) ( Figure 3f). Detailed breakdowns of isolates based on age, sex, location, source type, and specialty for ESKAPEE pathogens are listed in Table 1 and Figure 3.

**Table 1.** Total number of isolates by patient characteristics (age, gender, location, source type, specialty) focusing ESKAPEE

	Total no of isolates		Total no of isolates		Total no of isolates
**Gender**		**Source**		**Country**	
Male	335172	Blood	113064	United States	104180
Female	264083	Wound	92423	Spain	36831
Unknown	5863	Urine	85185	Italy	34191
**Age Group**		Sputum	73344	France	32813
19 to 64 Years	281191	Endotracheal aspirate	43635	Germany	29567
65 to 84 Years	209528	Abscess	42201	China	21736
85 and Over	44844	Others	41673	Mexico	17689
0 to 2 Years	28400	Bodily Fluids	31184	Belgium	15700
3 to 12 Years	20478	Bronchoalveolar lavage	30811	Canada	14390
13 to 18 Years	12814	Skin	13440	Portugal	12299
Unknown	7863	Ulcer	9020	Argentina	12030
**Patient Location**		Gall Bladder	6210	Israel	11304
Inpatient	284034	Decubitus	3635	Korea, South	10704
Outpatient	50219	Burn	2429	India	10579

### Exploratory data analysis

**Figure 4.**
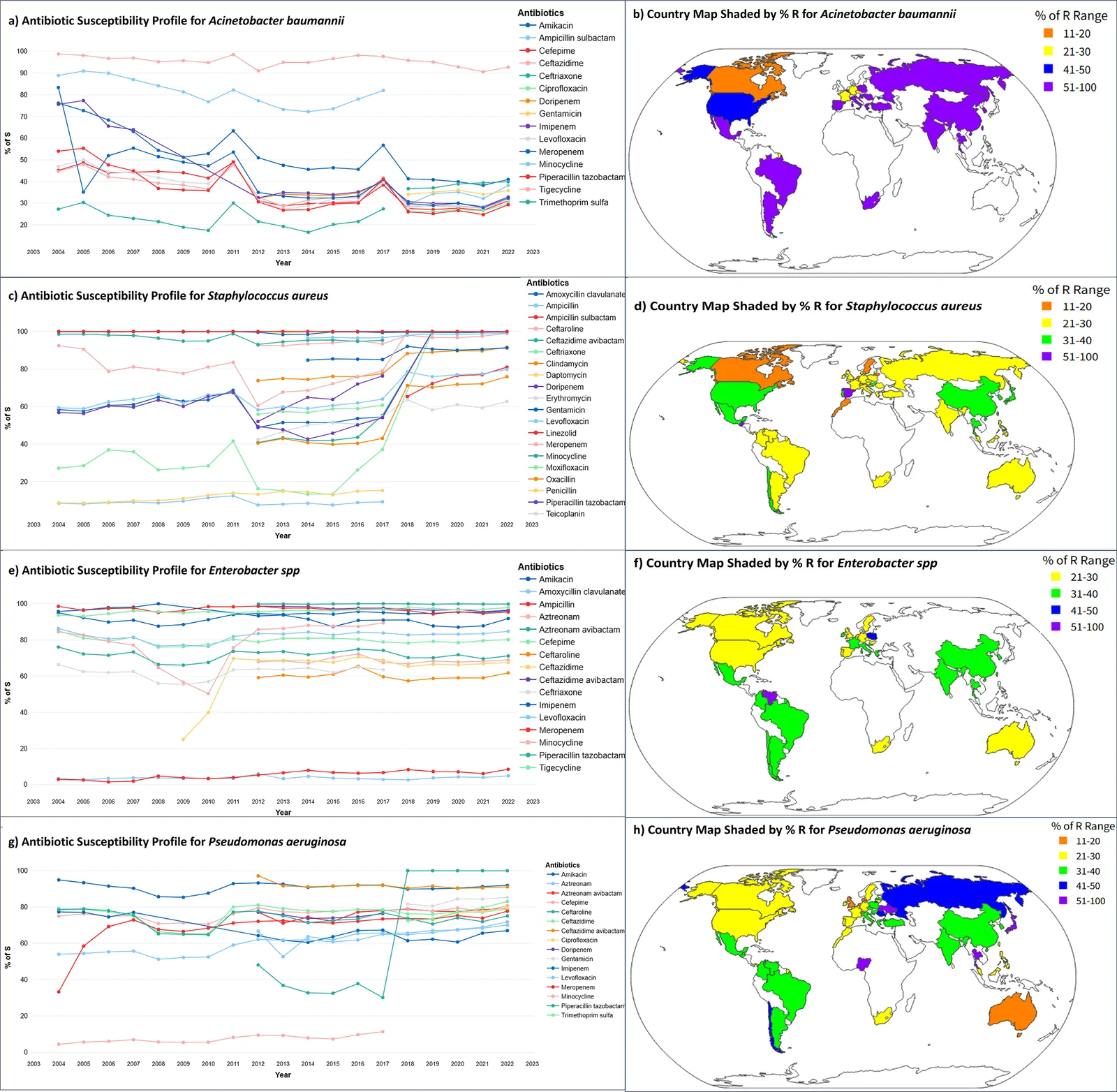
Time trend analysis of antibiotic susceptibility profile and Country wise resistance percentage of ESKAPPE Pathogens in MERIT Dashboard a&b) *Acinetobacter baumannii* c&d) *Staphylococcus aureus.* e&f ) *Enterobacter* species g&h *) Pseudomonas aeruginosa*.

For exploratory data analysis (EDA), the isolates were grouped by age, pathogens, and country, and MIC values for antibiotics. Resistance percentages were calculated over different age groups across different countries and organisms and visualized in plots under the “Antimicrobial Trend” tab. The X-axis represents different antibiotics, whereas the Y-axis represents the percentage of resistance (0 to 100%), and the different colored segments correspond to distinct age groups. Time trend analysis of Antimicrobial susceptibility profile of the few ESKAPEE pathogens and the country-wise resistance percentage were shown in Figure 4.

Key observations from EDA include:

• High (≥ 40%) and low (≤ 30%) resistance percentages across countries for some ESKAPEE pathogens. For instance, *A. baumannii* showed a higher percentage of resistance in countries such as Russia, India, Brazil, and Thailand, whereas Canada showed the lowest percentage of resistance (Figure b).
• *S. aureus* exhibited high resistance in China, the United States, and Mexico, whereas Australia, Sweden, and Canada had the lowest rates of resistance ( Figure 4d).
• *Enterobacter* species showed a high resistance percentage in Poland, whereas Australia, South Africa, and Sweden showed lower rates of resistance ( Figure 4f).
• High resistance rates for *P. aeruginosa* were observed in Ukraine, Romania, and Nigeria, whereas lower resistance rates were observed in Australia, United Kingdom, and the Netherlands ( Figure 4h).
• Time trend analysis showed a steady decline in susceptibility of *P. aeruginosa* to minocycline for *P. aeruginosa* over the past five years. Ampicillin resistance was notably high in *S. aureus* and *Enterobacter* species, and ceftriaxone showed a high resistance profile in *A. baumannii* and *Enterobacter* species, with significant increases observed in recent years ( Figure 4).

### Association rule mining in tracking shared MDR patterns

**Figure 5.**
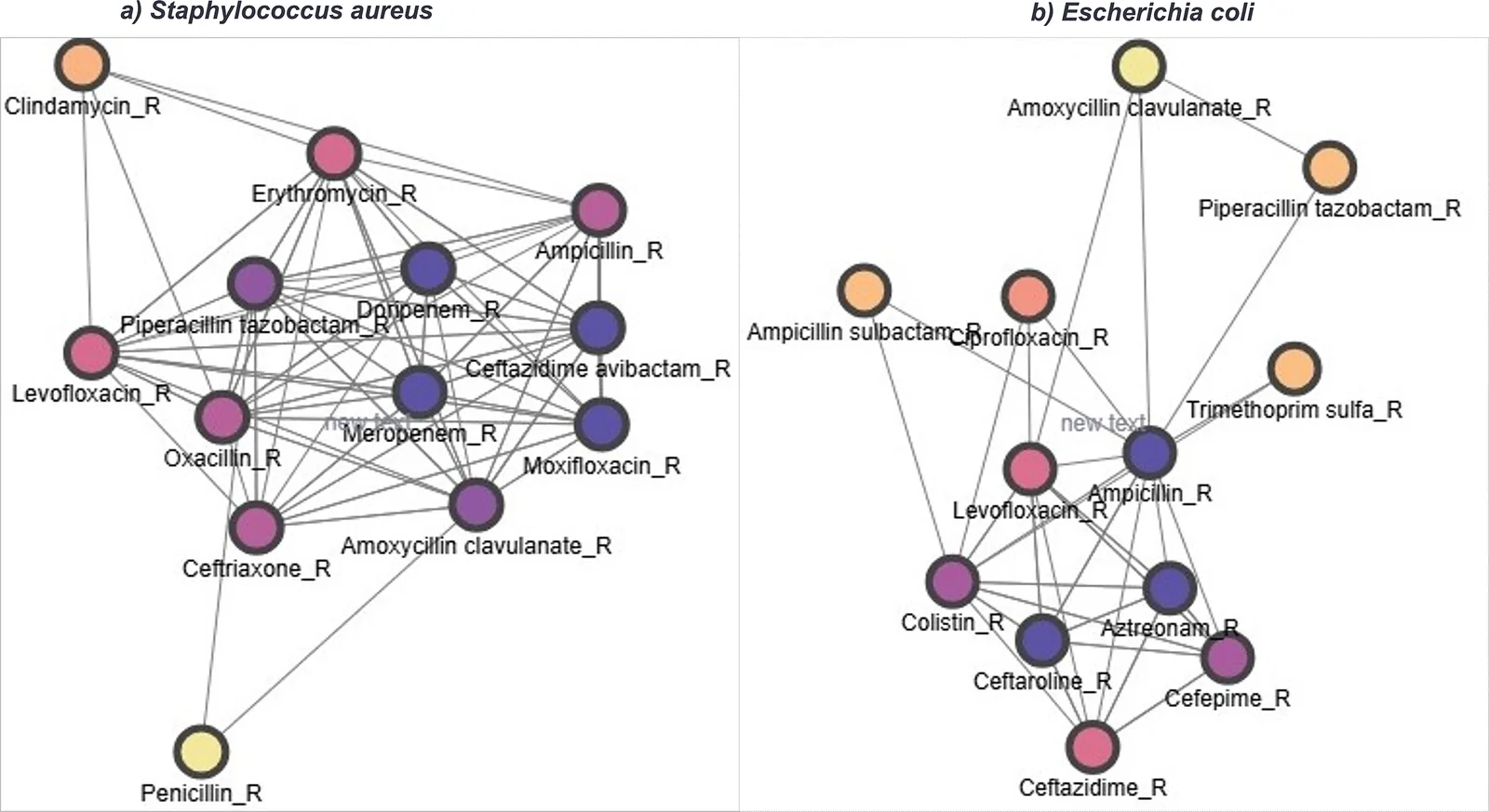
Resistance to Resistance pattern of *S. aureus* and *E. coli*.

**Figure 6.**
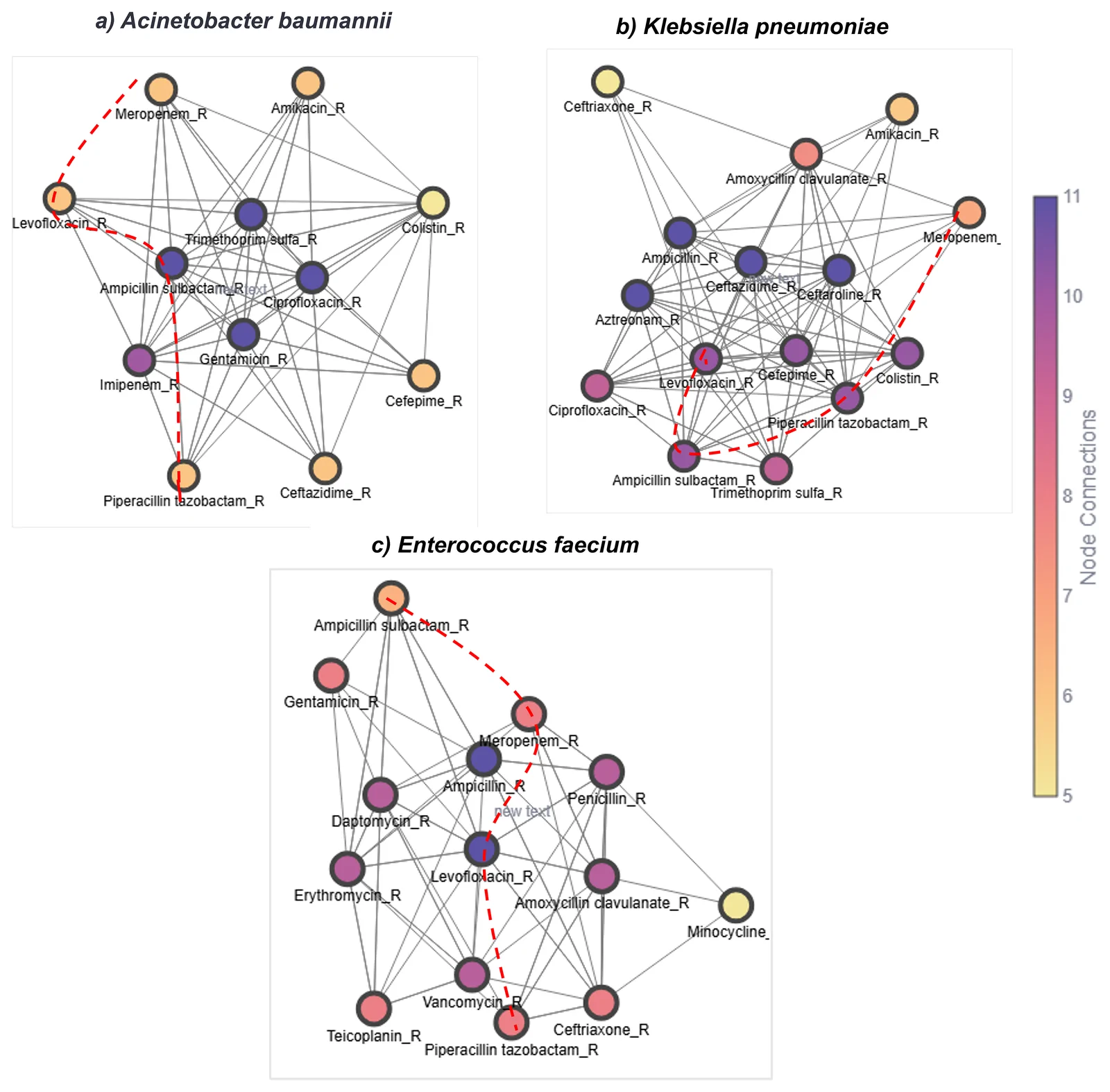
Shared multidrug resistance pattern observed in a) *Acinetobacter baumannii* b) *Klebsiella pneumoniae* c) *Enterococcus faecium.* Nodes represent antibiotics resistance and node color reflects the number of MDR connections (as indicated by the colour scale). The red dashed lines highlight antibiotics (Levofloxacin_R, Meropenem_R, Ampicillin sulbactam_R, and Piperacillin tazobactam_R) that exhibit shared resistance across all three pathogens.

Thus, the pattern of sMDR in the integrated antimicrobial surveillance datasets has been explored using the Apriori algorithm efficiently by implementing a quality measure selection pipeline, such as confidence, lift, support, and Zhang metrics. Antimicrobial surveillance data focusing on ESKAPEE pathogens exhibited infrequent resistant phenotypes to many of the tested antimicrobials, which in turn limited the utility of support as a pruning quality, thereby reducing the efficiency of the algorithm for finding the association rule. Therefore, we set the support value as >0.1, lift ≥ 2, and confidence ≥ 0.80, specific to the surveillance datasets for ESKAPEE pathogens. Based on these cutoffs, the following rules were generated for both “Resistant to Resistant” and “Resistant to Susceptible” Resistant to Susceptible’ patterns (Support = 0.1): Figure 5 and Figure 6 shown the “Resistance to Resistance rules generated for *S. aureus* and *E. coli* and sMDR observed in *A. baumannii, K. pneumoniae, E. faecium*

• *E. faecium* (n=1752)
• *S. aureus* (n=5158)
• *K. pneumoniae* (n=4857)
• *A. baumannii* (n=1621)
• *P. aeruginosa* (n=1475)
• *Enterobacter* species (n=3525)
• *E. coli* (n=3024)

*n = Total number of association rules

The total number of association rules provides insights into the evolution of antimicrobial resistance over time and enables the extraction of sMDR patterns across age groups and geographic locations. Some of the key observations in *S. aureus* revealed the possibility of cross-resistance to beta-lactamase inhibitor combinations (e.g., piperacillin-tazobactam) and other beta-lactams, such as ampicillin and ceftriaxone ( Figure 5a). Similarly, in *E. coli*, resistance to ceftazidime was frequently associated with colistin and aztreonam resistance ( Figure 5b). This pattern suggests the possibility of co-resistance, resulting in interconnected resistance patterns among antibiotics that target similar pathways. However, a relatively lower number of edges and nodes was observed between beta-lactam resistance and other antibiotic classes, indicating sMDR patterns involving multiple independent resistance mechanisms. For the personalized user interaction (interested organisms and resistance antibiotics) association query panel enables the display of significant association rules in “One-to-One” and “One-to-Many” search combinations. This approach has streamlined the interpretation of antimicrobial resistance trends in organisms and countries. Furthermore, sMDR patterns involving combinations of carbapenems, fluoroquinolones, and beta-lactamase inhibitors were observed in *A. baumannnii, K. pneumoniae and E. faecium* emphasizing the presence of MDR among these pathogens ( Figure 6). MERIT complements existing AMR surveillance efforts by extending isolate-level analyses based on association rule mining and network visualization of co-resistance patterns. In contrast to these resources, which primarily report aggregate resistance frequencies, MERIT enables systematic exploration of antibiotic co-occurrence and potential multidrug resistance (sMDR) patterns across specific organisms, geographic regions, years, and age groups. This functionality allows users to identify non-obvious resistance associations that may be masked in summary-level statistics, thereby offering additional analytical depth for hypothesis generation and surveillance interpretation.

## Discussion

The increasing global burden of AMR suggests the need for a robust surveillance strategy to identify and monitor the emerging resistance patterns ( Coque et al., 2023). In the current study, we developed the **“MERIT**” dashboard by integrating large-scale antimicrobial surveillance datasets (Pfizer-ATLAS and Venatorx-GEARS) over a period of 20 years (2004-2024) and applied association rule mining to identify shared multidrug-resistant (MDR) patterns among ESKAPEE pathogens. The MERIT Dashboard provides a user-friendly platform that could serve as a valuable tool by providing critical insights into resistance trends across different regions, pathogens, and drug classes by performing exploratory data analysis for the surveillance datasets and identifying key resistance trends. For example, across different geographical regions, India recorded the highest percentage of resistance for *E. coli* and *K. pneumoniae*, Russia for *A. baumannii*, and Poland for *Enterobacter* spp.; the United States, Germany, and Canada recorded the highest percentage of resistance against *Enterococcus faecium*, Ukraine, and Russia for *P. aeruginosa*, and China recorded against *S. aureus.* An increase in resistance to antibiotics such as ampicillin in *K. pneumoniae, S. aureus*, and *A. baumannii* indicates persistent challenges in combating these infections ( Denissen et al., 2022) and a further increase in resistance rates to other antibiotics such as fluoroquinolones, beta-lactams, and carbapenems, necessitating urgent intervention ( Mancuso et al., 2021). Interestingly, the steady decline in resistance to certain antibiotics, such as meropenem in *E. faecium* and doripenem in *P. aeruginosa*, may be attributed to recent therapeutic efforts. However, an increase in resistance to imipenem ( *K. pneumoniae, A. baumannii, Enterobacter spp.*) and meropenem ( *S. aureus, A. baumannii, Enterobacter spp.*) suggests an ongoing rise in carbapenem resistance, particularly in gram-negative pathogens. This trend aligns with global reports highlighting the rapid spread of carbapenemase-producing *Enterobacterales* (CPE), often linked to OXA- and NDM-type carbapenemases ( Han et al., 2020; Lee et al., 2022; WHO, 2023). Understanding these evolving resistance trends is essential to facilitate timely decision-making for a broad range of stakeholders, including clinicians, researchers, public health officials, and policymakers.

A key strength of the MERIT dashboard is that it provides a network-based visualization of shared antibiotic resistance patterns across various dimensions, including geographical regions, age groups, and temporal trends. sMDR is a complex phenomenon of MDR that poses a major threat to human health in this century compared to pathogens with single drug resistance ( Choi et al., 2024). MDR refers to a resistance mechanism when a single organism is resistant to multiple drugs, whereas sMDR is evident when multiple species share the same resistance mechanisms, often due to horizontal gene transfer (HGT) in bacteria. sMDR leads to outbreaks of untreatable infections, especially in hospital settings, where resistance genes spread through plasmids, transposons, or integrons. Thus, in the current study, we aimed to identify such sMDR patterns from antimicrobial susceptibility testing (AST) results by analyzing phenotypic resistance data. Surveillance datasets contain thousands of isolates tested against multiple antimicrobials, leading to high-dimensional data that require manual feature selection and do not scale well when simultaneously exploring interactions among multiple antimicrobials. However, traditional statistical methods such as logistic regression and principal component analysis (PCA) exhibit significant limitations in detecting hidden multivariate relationships, co-occurrence, or sMDR patterns from large-scale antimicrobial susceptibility testing (AST) datasets. To overcome these limitations, we employed association rule mining (ARM), particularly the Apriori algorithm, which can identify frequently co-occurring resistance between different antimicrobials without requiring predefined hypotheses. In the study conducted by Cazer et al. (2019), key associations between antimicrobial drugs were identified, and temporal trends and differences in MDR patterns were examined. Association rule mining has also been applied to study MDR Prevalence and visualize the associations between resistance patterns ( Mosaddegh et al., 2024; Cazer et al., 2021; Liang et al., 2021). This proves that association rule mining is an effective tool for identifying patterns of MDR within antimicrobial susceptibility testing data and for evaluating the statistical and biological significance of the patterns. However, this study did not apply any statistical testing correction; instead, effect-size-based thresholds (support, confidence, lift, and Zhang’s metric) were used to reduce spurious associations. Consequently, some false-positive rules may still exist, particularly in datasets generating large numbers of associations. Furthermore, unlike regression-based models, the Apriori algorithm efficiently processes large itemsets of antimicrobial resistance phenotypes to reveal association rules. For example, we ranked significant MDR patterns among carbapenem, fluoroquinolone, and aminoglycoside resistance, along with measures such as support (frequency of pattern), confidence (reliability of association), and lift (strength of association). Thus, association rule mining offers a robust framework for predicting emerging MDR patterns, which can aid in the development of targeted antimicrobial stewardship programs. For example, in country-wise rule mining, India’s resistance to aztreonam is frequently associated with resistance to Cefepime, Ampicillin, Ceftazidime, Levofloxacin, Ceftaroline, and Ciprofloxacin. Similarly, it helps unveil the resistance and susceptibility trends of antibiotics. For example, in India, if resistance to Colistin + Ampicillin is observed, then the choice of the susceptible drug could be Meropenem and Ceftazidime avibactam. Such insights into resistance and resistance susceptibility patterns can guide stakeholders in optimizing antimicrobial therapy and potentially reduce the prescription of broad-spectrum antibiotics. Furthermore, this could lead to the discovery of new MDR patterns, which were not evident through traditional methods. These findings have laid the groundwork for future research on developing novel therapeutic strategies to combat MDR bacteria and assist in clinical decision-making, infection prevention, cost management, and public health using the data-driven approach. The limitation of this work is that association mining was performed exclusively on MIC-based phenotypic data, without incorporating genomic information. Consequently, the present dataset does not allow us to determine whether these shared resistance traits arise from horizontal gene transfer or from independent evolutionary events.

### Ethics and Consent

Ethical approval and consent were not required for this study. However, the study proposal underwent review and approval by Vivli prior to data access being granted.

### Dashboard availability

**Weblink:** https://merit.sastra.edu/

### Declaration of generative AI and AI-assisted technologies in the writing process

During the preparation of this work, the authors used ChatGPT-4 in order to improve readability. After using this tool, the authors reviewed and edited the content as needed, and take full responsibility for the content of the published article.

## Data Availability

ATLAS (Antimicrobial Testing Leadership and Surveillance) dataset, conducted by Pfizer and GEARS (Global Evaluation of Antimicrobial Resistance via Surveillance) conducted by Venatorx, were used in this study and are available on request to the Vivli AMR database via https://amr.vivli.org. These datasets are part of the Vivli-AMR Register and to access these datasets, interested researchers must follow the detailed dataset request procedure as outlined in the Vivli-AMR Register documentation. This procedure includes searching for datasets using keywords and browsing functionalities on the Vivli-AMR platform, completing the Data Request Form, and obtaining approval from the relevant Data Contributors. All data provided through the Vivli-AMR Register are subject to the data sharing agreements and policies set forth by individual data contributors and the platform. For further details on the process of requesting datasets, please refer to the Vivli-AMR Register’s official documentation or contact the Vivli support team at amr@vivli.org. The data used in the current study have not been shared via the repository. **Source code available from:** https://github.com/bic-sastra/MERIT_Dashboard **Archived software available from:** https://doi.org/10.5281/zenodo.15393861 **License:** GNU General Public License v3.0 [OSI approved open license software is under]
