# Supplementary material for: Exploring the evolution of multidrug resistance patterns in ESKAPEE pathogens using association mining: Key to antibiotic stewardship?
Source: Wellcome Open Res. 2026 Jul 17;10:461. Originally published 2025 Aug 22. [Version 3] doi: 10.12688/wellcomeopenres.24103.3 (PMC13560748; doi:10.12688/wellcomeopenres.24103.3)
Supplement: Peer review history

# Reviewer report: Reviewer response for version 3

**DOI:** 10.21956/wellcomeopenres.29978.r161300

**Published:** 2026-08-05

**Authors:** Pownraj Brindangnanam

**Affiliations:** 1 Pondicherry University, Kalapet, Pondicherry, India

The authors have satisfactorily addressed the concerns raised in my previous review report.

Is the work clearly and accurately presented and does it cite the current literature?

Yes

If applicable, is the statistical analysis and its interpretation appropriate?

Partly

Are all the source data underlying the results available to ensure full reproducibility?

Partly

Is the study design appropriate and is the work technically sound?

Partly

Are the conclusions drawn adequately supported by the results?

Partly

Are sufficient details of methods and analysis provided to allow replication by others?

Partly

Reviewer Expertise:

AMR, Bioinformatics, CADD, ML

I confirm that I have read this submission and believe that I have an appropriate level of expertise to confirm that it is of an acceptable scientific standard.

# Reviewer report: Reviewer response for version 2

**DOI:** 10.21956/wellcomeopenres.28259.r149736

**Published:** 2026-03-28

**Authors:** Rasha Abdelsalam Elshenawy

**Affiliations:** 1 University of Hertfordshire, Hatfield, England, UK

I have reviewed the revised manuscript (version 2) along with the authors' point-by-point responses to both prior reviewers. I acknowledge that the authors have made a genuine effort to address the concerns raised, and several targeted improvements are evident. However, a number of substantive issues remain insufficiently resolved, and I outline these below.

 The authors have meaningfully improved the manuscript in several respects. The Methods section now explicitly clarifies that two different support thresholds were used (≥0.3 for robust cross-year/age/country patterns and >0.1 for sparser organism-specific subsets), which resolves the confusion flagged by the previous reviewer. The addition of explicit limitations regarding the absence of formal multiple-testing correction, data heterogeneity, and reproducibility constraints tied to the data-use agreement is appropriate and appreciated. The Discussion has been expanded to better contextualise sMDR findings and to acknowledge that temporal comparisons should be treated descriptively rather than as strict statistical comparisons. The deposition of code on GitHub and the archiving on Zenodo are commendable steps toward reproducibility.

**Important issues need to be addressed:**

**1. Clinical relevance of antibiotic-pathogen pairings (Reviewer 1, major concern)**

 The authors acknowledged that microbiology/infectious disease expertise was absent from the team and committed to incorporating it in future iterations. However, this limitation has not been addressed in the current version. Clinically implausible associations — such as meropenem resistance in *Enterococcus faecium* or *S. aureus*, where carbapenems are not clinically indicated — remain present in the association outputs without adequate biological contextualisation or filtering. Given that the stated aim is to support antibiotic stewardship, this is not a minor issue. At minimum, the authors should add a clearly visible disclaimer in both the manuscript and the dashboard itself, warning users that some rules may reflect testing artefacts or clinically irrelevant pairings, and recommending clinical review before acting on any rule.

**2. False discovery and statistical rigour**

 The authors acknowledged that no formal false discovery rate (FDR) correction was applied, and they committed to including this as a limitation. This is now stated in the text, which is acceptable for an exploratory study. However, the manuscript should be more explicit that the rules are hypothesis-generating only, and the abstract and conclusions should avoid language that implies clinical actionability without this caveat. The current conclusion — "our findings from the data challenge could aid healthcare professionals in making informed decisions regarding antibiotic use", overstates the readiness of these findings for clinical application and should be tempered.

**3. Validation of sMDR patterns (Q9)**

 The authors confirmed that no validation against genomic data, known resistance genes, or mobile genetic elements was performed. This is a significant limitation given that the central claim of the study is the identification of *shared* multidrug resistance patterns, a concept that inherently implies a shared mechanism. The authors acknowledge they cannot distinguish horizontal gene transfer from convergent evolution, which is appropriate, but the manuscript should more clearly frame all findings as phenotypic co-occurrence patterns rather than confirmed shared resistance mechanisms. The terminology "sMDR" throughout the manuscript implies mechanistic sharing, which is not supported by the data.

**4. Threshold justification and sensitivity analysis (Q3)**

 While the authors have now clarified that thresholds were chosen pragmatically based on dataset size, no formal sensitivity analysis was conducted. This is explicitly acknowledged, but a brief supplementary analysis showing how the number and strength of rules change under alternative threshold combinations (e.g., support = 0.05, 0.1, 0.2) for at least one representative organism would substantially strengthen confidence in the chosen parameters. The reviewer appreciates the authors' honesty here but encourages this as a minimal addition.

**5. Dashboard usability limitations (Q13, Q14, Q15)**

 The authors acknowledged several dashboard usability issues, including the absence of actionable guidance when "No rules found" is returned, the lack of active tab highlighting, and the absence of sample-size warnings for sparse country-specific queries, and stated these are planned for future upgrades. While it is understandable that not all enhancements can be implemented in this revision cycle, it would strengthen the manuscript if these limitations were clearly enumerated in a dedicated "Limitations of the MERIT dashboard" subsection, so that users of the current version are properly informed.

**6. Reproducibility under data-use restrictions (Q6)**

 The code has been deposited on GitHub and Zenodo, which is positive. However, since the underlying data cannot be redistributed under the Vivli data-use agreement, independent replication of the specific results is not fully possible. The authors should explicitly acknowledge this in the manuscript under data availability or limitations, noting that the code can be applied to independently requested Vivli datasets but that exact result reproduction requires individual data access approval from Vivli.

**7. Temporal trend interpretation and breakpoint evolution (Q7)**

 The authors confirmed that year-specific CLSI breakpoints were used and stated that specific yearly versions will be cited in the revised methods. However, these citations do not appear to have been added in the version 2 manuscript provided for review. Including these citations would be a straightforward but important step for transparency.

**Minor Points**

- The abstract conclusion could be more carefully worded to reflect the exploratory nature of the work, particularly avoiding phrasing that implies direct clinical utility without validation.
- The paper's discussion of MERIT's unique value proposition relative to ECDC EARS-Net, Pfizer ATLAS portal, and WHO GLASS (Q16) has been addressed in the author response but does not appear to be substantively incorporated into the Discussion section of the revised manuscript. A short paragraph on this distinction would benefit readers.
- The authors note that ChatGPT-4 was used to improve readability, which is disclosed appropriately. Reviewers should be aware that some passages may benefit from further technical editing to ensure precision of language, particularly in the Methods and Discussion.

**Summary**

 This manuscript addresses a genuinely important problem in AMR surveillance and the MERIT dashboard represents a useful contribution to the field. The authors have responded constructively to prior reviewers and made meaningful revisions. However, the study falls short of full approval without reservations, primarily because: (1) clinically implausible pathogen-antibiotic pairings have not been filtered or adequately flagged; (2) the language around sMDR implies mechanistic sharing that the data cannot support; and (3) several stated future improvements are necessary for responsible clinical use of the dashboard. I recommend **approved with reservations**, contingent on the authors addressing points 1–3 above in the manuscript text and adding the missing CLSI breakpoint citations.

**Competing Interests:** No competing interests.

Is the work clearly and accurately presented and does it cite the current literature?

Yes

If applicable, is the statistical analysis and its interpretation appropriate?

Partly

Are all the source data underlying the results available to ensure full reproducibility?

Partly

Is the study design appropriate and is the work technically sound?

Partly

Are the conclusions drawn adequately supported by the results?

Partly

Are sufficient details of methods and analysis provided to allow replication by others?

Partly

Reviewer Expertise:

Antimicrobial resistance, infectious diseases, clinical microbiology, antibiotic stewardship

I confirm that I have read this submission and believe that I have an appropriate level of expertise to confirm that it is of an acceptable scientific standard, however I have significant reservations, as outlined above.

# Response

**Published:** 2026-07-03

**Authors:** Suma Mohan

**Affiliations:** Division of Bioinformatics, SASTRA Deemed to be University, Thanjavur, Tamilnadu, India

**Reviewers’ response ** We thank the reviewer for reviewing our manuscript entitled "Exploring the evolution of multidrug resistance patterns in ESKAPEE pathogens using association mining: Key to antibiotic stewardship?" and viewer response and for providing helpful comments. With this, we enclosed a point-by-point response to the reviewer’s concerns for publication in your esteemed journal. We believe that we did our best to remove all the ambiguities and made changes that clearly improved our manuscript, which We have uploaded alongside this document.  

1. **Clinical relevance of antibiotic-pathogen pairings (Reviewer 1, major concern)**
** The authors acknowledged that microbiology/infectious disease expertise was absent from the team and committed to incorporating it in future iterations. However, this limitation has not been addressed in the current version. Clinically implausible associations — such as meropenem resistance in *Enterococcus faecium* or *S. aureus*, where carbapenems are not clinically indicated — remain present in the association outputs without adequate biological contextualisation or filtering. Given that the stated aim is to support antibiotic stewardship, this is not a minor issue. At minimum, the authors should add a clearly visible disclaimer in both the manuscript and the dashboard itself, warning users that some rules may reflect testing artefacts or clinically irrelevant pairings, and recommending clinical review before acting on any rule.**

**Response: **We fully agree and thank the reviewer for this important observation and agree that certain antibiotic-pathogen associations identified through association rule mining may not necessarily correspond to clinically relevant treatment combinations. Since the analysis was performed only based on the available phenotypic susceptibility data in the Vivli data source, some association rules may reflect surveillance practices rather than true clinically associated relationships. We have now added a clearly visible disclaimer in the Results section of the manuscript as follows: “Association rules identified in the current study may not necessarily correspond to clinically relevant treatment combinations. Because the analysis was performed on phenotypic susceptibility testing data available in the vivli data where some rules may reflect clinically irrelevant antibiotic-pathogen pairings. Thus, association rules should be interpreted as data-driven resistance co-occurrence patterns and it is strongly recommended to review by a clinician or infectious disease experts before acting on any rule generated by MERIT dashboard.” We believe that the addition of this statement as disclaimer explicitly makes a meaningful and responsible step towards the usage of the dashboard outputs.

1. **False discovery and statistical rigour**
** The authors acknowledged that no formal false discovery rate (FDR) correction was applied, and they committed to including this as a limitation. This is now stated in the text, which is acceptable for an exploratory study. However, the manuscript should be more explicit that the rules are hypothesis-generating only, and the abstract and conclusions should avoid language that implies clinical actionability without this caveat. The current conclusion — "our findings from the data challenge could aid healthcare professionals in making informed decisions regarding antibiotic use", overstates the readiness of these findings for clinical application and should be tempered.**

**Response: **Thank you for this important constructive suggestion. We agree that, the identified association rules as hypothesis-generating rather than clinically validated findings. Accordingly, we revised the Abstract, Discussion and Conclusion sections of the manuscript and explicitly stated the following limitations. a) No actionable guidance when "No rules found"; (b) No active tab highlighting; (c) No sample-size warnings for sparse queries; (d) clinically implausible pairings not filtered; (e) no FDR correction; (f) No genomic validation; (g) Data access restrictions limiting exact reproducibility.  

1. **Validation of sMDR patterns (Q9)**
** The authors confirmed that no validation against genomic data, known resistance genes, or mobile genetic elements was performed. This is a significant limitation given that the central claim of the study is the identification of *shared* multidrug resistance patterns, a concept that inherently implies a shared mechanism. The authors acknowledge they cannot distinguish horizontal gene transfer from convergent evolution, which is appropriate, but the manuscript should more clearly frame all findings as phenotypic co-occurrence patterns rather than confirmed shared resistance mechanisms. The terminology "sMDR" throughout the manuscript implies mechanistic sharing, which is not supported by the data.**

**Response: **We thank the reviewer for this important observation. We respectfully clarify that the terminology "shared multidrug resistance" was adopted to describe multidrug resistance patterns identified through association rule mining of phenotypic antimicrobial susceptibility data, as used by Cazer et al. (2019)., We employed a similar approach to characterise shared resistance patterns among ESKAPEE pathogens using phenotypic MIC data, Apriori association rule mining, and network decomposition without genomic validation. Consistent with prior study, we use sMDR to denote co-occurrence patterns identified through association rule filtering and not a confirmed shared mechanism. However, we agree that the terminology horizontal gene transfer used in the Introduction part was not evaluated in the current study and overstates what phenotypic data alone can support. The identified associations should be interpreted as resistance co-occurrence patterns rather than direct evidence of shared molecular mechanisms. To improve clarity and avoid overinterpretation, we have revised the manuscript throughout to explicitly define sMDR as phenotypic multidrug resistance co-occurrence patterns identified through association rule mining. Additional text has been added to the Discussion and Limitations sections emphasizing that genomic validation was beyond the scope of the present study and that future analyses will be necessary to determine the biological mechanisms underlying the observed patterns.

1. **Threshold justification and sensitivity analysis (Q3)**

While the authors have now clarified that thresholds were chosen pragmatically based on dataset size, no formal sensitivity analysis was conducted. This is explicitly acknowledged, but a brief supplementary analysis showing how the number and strength of rules change under alternative threshold combinations (e.g., support = 0.05, 0.1, 0.2) for at least one representative organism would substantially strengthen confidence in the chosen parameters. The reviewer appreciates the authors' honesty here but encourages this as a minimal addition.

**Response: **We thank the reviewer and understand its scientific value. We agree that sensitivity analysis across alternative support thresholds would substantially strengthen the work by providing additional insight into the robustness of the identified association rules. We performed a targeted sensitivity analysis on the sMDR pattern presented in Figure 6. (meropenem, levofloxacin, ampicillin/sulbactam, piperacillin/tazobactam resistance), which is shared across *A. baumannii, K. pneumoniae*, and *E. faecium *by systematically varying support (0.1-0.5) and lift (> 1.5 and >2) thresholds while holding confidence and Zhang's metric fixed at the values used in the main analysis. The resulting resistance networks with varying thresholds for the three organisms are presented in Supplementary Figure S1. As expected, lower support thresholds generated a larger number of association rules, whereas higher thresholds retained fewer, stronger associations. For the specific sMDR pattern in Figure 6, we found that tightening the support and lift thresholds led to organism-specific degradation of the full four-drug pattern: Ampicillin/sulbactam resistance was the most threshold-sensitive component, dropping out earliest in *A. baumannii* and *K. pneumoniae*, while piperacillin/tazobactam and levofloxacin co-resistance association represents a comparatively robust shared signal across these three ESKAPEE pathogens. We have incorporated these findings into the Result part. We believe this targeted sensitivity analysis substantively addresses the reviewer's concern about threshold robustness.  

1. **Dashboard usability limitations (Q13, Q14, Q15)**

The authors acknowledged several dashboard usability issues, including the absence of actionable guidance when "No rules found" is returned, the lack of active tab highlighting, and the absence of sample-size warnings for sparse country-specific queries, and stated these are planned for future upgrades. While it is understandable that not all enhancements can be implemented in this revision cycle, it would strengthen the manuscript if these limitations were clearly enumerated in a dedicated "Limitations of the MERIT dashboard" subsection, so that users of the current version are properly informed. **Response **: Thank you for this constructive suggestion. We have added a dedicated "Limitations" subsection to the manuscript to explicitly state the known limitations to the users of the MERIT dashboard. Limitations The identified sMDR patterns were derived from phenotypic antimicrobial susceptibility data and no genome analyses were performed. So, the observed associations should be inferred as phenotypic co-occurrence patterns rather than confirmed shared molecular resistance mechanisms. (B) No actionable guidance when "No rules found"; (C) No active tab highlighting is available in the dashboard; (D) No sample-size warnings for sparse queries; (E) clinically implausible pairings not filtered and requires experts’ interpretation; (F) no FDR correction was applied; (G) Data access restrictions associated with the Vivli AMR datasets limit exact reproducibility of the reported results. We believe that this dedicated subsection enumerates the dashboard usability issues raised in Q13-Q15.    

1. **Reproducibility under data-use restrictions (Q6)**

The code has been deposited on GitHub and Zenodo, which is positive. However, since the underlying data cannot be redistributed under the Vivli data-use agreement, independent replication of the specific results is not fully possible. The authors should explicitly acknowledge this in the manuscript under data availability or limitations, noting that the code can be applied to independently requested Vivli datasets but that exact result reproduction requires individual data access approval from Vivli. **Response: **Thank you for highlighting this. We have revised the Data Availability section and now added a dedicative “Reproducibility” subsection to explicitly state that an independent data access approval from Vivli is recommended for exact reproduction of the results. We believe this directly addresses the reviewer's request and provides appropriate transparency regarding reproducibility.  

1. **Temporal trend interpretation and breakpoint evolution (Q7)**

The authors confirmed that year-specific CLSI breakpoints were used and stated that specific yearly versions will be cited in the revised methods. However, these citations do not appear to have been added in the version 2 manuscript provided for review. Including these citations would be a straightforward but important step for transparency. **Response:** We apologise for this oversight. Phenotype assignment from MIC values using year-specific CLSI breakpoints were performed specifically for isolates from the 2014–2021 period where MIC data was available but R/S/I phenotype labels were missing; isolates from other years with missing phenotype labels were excluded rather than assigned using the corresponding annual CLSI breakpoint criteria. We have now added the specific CLSI yearly document citations in the Methods section under "Dataset cleaning and wrangling. The corresponding MIC breakpoints for each organism across this period are provided in the newly added Supplementary Table S2. These references have been added to improve transparency and facilitate interpretation of temporal analyses.   We are grateful to the reviewer for the thorough and expert evaluation, and for acknowledging the genuine contribution of the MERIT dashboard to AMR surveillance. We have addressed all major and minor points raised in this review.

**Minor points**  

- **The abstract conclusion could be more carefully worded to reflect the exploratory nature of the work, particularly avoiding phrasing that implies direct clinical utility without validation.**

**Response:** We appreciate the reviewer’s suggestion and have revised the Abstract conclusion as to better reflect the exploratory nature of the work and to remove any language implying direct clinical utility without validation.  

- **The paper's discussion of MERIT's unique value proposition relative to ECDC EARS-Net, Pfizer ATLAS portal, and WHO GLASS (Q16) has been addressed in the author response but does not appear to be substantively incorporated into the Discussion section of the revised manuscript. A short paragraph on this distinction would benefit readers.**

**Response: **We thank the reviewer for this recommendation. A new paragraph has been added to the Discussion section describing the relationship between MERIT and existing antimicrobial resistance surveillance platforms, including EARS-Net, ATLAS, and GLASS. The revised text highlights that MERIT is intended to complement existing surveillance resources by enabling exploratory association rule mining and resistance co-occurrence pattern discovery rather than replacing established surveillance systems.  

- **The authors note that ChatGPT-4 was used to improve readability, which is disclosed appropriately. Reviewers should be aware that some passages may benefit from further technical editing to ensure precision of language, particularly in the Methods and Discussion.**

 
**Response:** We thank the reviewer for this observation. The manuscript has undergone an additional round of technical editing to improve clarity, particularly within the Methods and Discussion sections.

# Reviewer report: Reviewer response for version 1

**DOI:** 10.21956/wellcomeopenres.26591.r136678

**Published:** 2025-11-13

**Authors:** Hamad Abdel Hadi

**Affiliations:** 1 Medical College, Qatar University, Doha, Doha, Qatar

Saksika Rajendran et al submitted: Exploring the evolution of multidrug resistance patterns in ESKAPEE pathogens using association mining: Key to antibiotic stewardship

 The idea is very excellent: extracting surveillance data from major AMR data bases and examine them for trend changes for the ESKAPE pathogens (both geographical and microbiological variations) using their expertise in IT and bioinformatics through data mining

 The major flaw of the study methods and design, they did not include expertise in microbiology/infection to guide them for data interpretation (which antimicrobials is effective and logical for which pathogens i.e. meropenem and MRSA and Enterococci) That led to analysis output that will not conform to speciality standards and meaningful results. If that is rectified the study will be useful

Is the work clearly and accurately presented and does it cite the current literature?

Yes

If applicable, is the statistical analysis and its interpretation appropriate?

Yes

Are all the source data underlying the results available to ensure full reproducibility?

Yes

Is the study design appropriate and is the work technically sound?

Yes

Are the conclusions drawn adequately supported by the results?

No

Are sufficient details of methods and analysis provided to allow replication by others?

No

Reviewer Expertise:

Antimicrobial Reistance

I confirm that I have read this submission and believe that I have an appropriate level of expertise to confirm that it is of an acceptable scientific standard, however I have significant reservations, as outlined above.

# Response

**Published:** 2025-12-17

**Authors:** Suma Mohan

**Affiliations:** Division of Bioinformatics, SASTRA Deemed to be University, Thanjavur, Tamilnadu, India

Thank you very much for reviewing our manuscript entitled "Exploring the evolution of multidrug resistance patterns in ESKAPEE pathogens using association mining: Key to antibiotic stewardship?". Thank you for this important observation. We agree that microbiology and infectious-disease expertise are essential for interpreting AMR surveillance outputs and ensuring that the associations examined are clinically meaningful. Our team primarily approached the problem from a data-science and bioinformatics perspective, and we acknowledge that the absence of direct infectious-disease/microbiology expert involvement is a limitation of the current version of the work. However, we followed standard CLSI breakpoint guidelines, pathogen-specific recommended panels, and cross-checked associations against published literature to avoid biologically implausible interpretations. We appreciate the reviewer’s suggestion and will incorporate microbiology domain expertise in the next iteration to refine pathogen-antibiotic pairing, add biological context filters, and improve clinical interpretability.

# Reviewer report: Reviewer response for version 1

**DOI:** 10.21956/wellcomeopenres.26591.r136686

**Published:** 2025-11-12

**Authors:** Pownraj Brindangnanam

**Affiliations:** 1 Pondicherry University, Kalapet, Pondicherry, India

**QUESTIONS FOR AUTHORS (Major revision)**

**Major Concerns:**

 Q1. The manuscript identifies sMDR patterns across species (Figure 6), but the dashboard only allows single-organism queries. Author should clarify: (a) Was Figure 6 created by running separate analyses per organism and manually comparing networks? (b) Why can't users perform this cross-species analysis directly in the dashboard? (c) How do author computationally define "shared" patterns beyond visual overlap?

 Q2. With thousands of rules generated per organism (e.g., A. baumannii n=3,132), how author control for false discovery rate?. Are multiple testing corrections applied?

 Q3. Author mentioned support >0.1 due to infrequent resistance. However: (a) What quantitative evidence supports this threshold? (b) Was sensitivity analysis performed? (c) Why do many queries still return "No rules found"? (d) Paper mentions both support ≥0.3 and >0.1 - which was used and why the discrepancy?

 Q4. The Methods mention that isolates without MIC values were excluded but those with MIC values, yet a missing phenotype were manually assigned. How many of the isolates had complete testing profiles for all the antibiotics tested? How might differential testing across organisms-for example, organism A being tested against 20 antibiotics and organism B tested against 15-affect comparability of association rules? Can incomplete testing matrices lead to biased discovery of co-resistance patterns or spurious sMDR patterns?

 Q5. Many surveillance programs utilize risk-based testing strategies in which carbapenems are tested only when resistance to first-line agents is identified. Might this conditional testing generate spurious associations, where carbapenem resistance seems associated with other resistances just because both are preferentially tested in already-resistant isolates? How do author separate real biological co-resistance from testing ascertainment bias?

**Reproducibility & Transparency:**

 Q6. Can other researchers reproduce author’s association rule mining results? Is the complete analysis code openly available, such as a GitHub repository? Are the specific data preprocessing steps, MIC breakpoint assignments, and phenotype categorization rules for the Pfizer-ATLAS and Venatorx-GEARS datasets fully documented to enable independent replication?

 Q7. For the MIC breakpoint interpretation, which guidelines were followed? For example, CLSI, EUCAST? Were year-specific versions utilized, given data spans 2004-2022, a period when breakpoints changed for key antibiotics? How does the evolution of these guidelines affect temporal trend analysis?

 Q8. How many the isolates contributed to author’s result for Switzerland+A. baumannii+Tigecycline+Year 2022? The interface says, "No rules extracted". Is it because of lack of data or too strict thresholds of author’s parameters? What sample size would author recommend in general for a reliable production of rules for an organism-country-year-antibiotic combination? Should the dashboard show some kind of data sufficiency warning or sample size indication before a user starts to invest time into a query?

**Validation:**

 Q9 Have author validated identified sMDR patterns against known resistance mechanisms or genetic data? More specifically, do the co-resistance patterns highlighted in Figure 6 (levofloxacin-meropenem-ampicillin/sulbactam-piperacillin/tazobactam across A. baumannii, K. pneumoniae, and E. faecium) correspond to known resistance genes, plasmids, integrons, or mobile genetic elements? How would author differentiate true sMDR from horizontal gene transfer versus independent evolution to a similar resistance phenotype?

 Q10. What is the negative predictive value and the confidence interval for the "Resistant to Susceptible" (R→S) rules discussed in the Discussion, such as "if resistant to Colistin+Ampicillin, then susceptible to Meropenem"? How reliable are these rules in guiding clinical antibiotic selection? Should the dashboard show prediction intervals or uncertainty estimates to inform appropriate clinical use?

 Q11. The interface has "Select Year: 2022" for discrete year selection. Are users able to query how the association rules themselves have varied over time? For instance, can users visualize that "levofloxacin→meropenem had lift=1.5 in 2004 but lift=2.8 in 2022"? Is there an ability in place to track the emergence, strengthening, or weakening of specific resistance associations across the period of surveillance?

**Dashboard Usability & Design:**

 Q12. The network graphs have different numbers of rules: E. faecium with n=1,752, S. aureus with n=5,158, and K. pneumoniae with n=4,857. How can these complex networks be navigated within the dashboard? Are there active filtering options, such as "show only rules with lift >3" or "hide nodes with <5 connections", a search function, or even hierarchical layout to improve interpretability?

 Q13. In the case of the dashboard showing "No rules found, try with new parameters," there is no actionable guidance for the user. Please provide specific suggestions regarding: a) which parameters to change (support versus lift versus minimum support); b) which direction to go; and c) whether the problem is not enough data or too restrictive thresholds. Consider adding context-sensitive help or suggestion algorithms for parameters.

 Q14. All tabs appear with the same styling-white background and blue text-regardless of which page is active. In the absence of any visual indicators like highlighting, a different color for the background, bolding of text, or an 'active' state indicator, how is a user supposed to maintain spatial awareness regarding their current location while traversing between the six tabs?

 Q15. The USA contributed 17% of the isolates while many countries contributed < 1%. How does this geographic imbalance affect the reliability of country-specific association rules? Should results be presented with sample-size-weighted confidence or should the dashboard display warnings when country-specific queries fall below recommended thresholds?

**Context & Broader Impact**

 Q16. How does MERIT compare functionally and analytically to other established AMR surveillance dashboards and tools, for example, the Pfizer ATLAS online portal, ECDC EARS-Net, ResistanceMap, WHO GLASS? What is MERIT's unique value proposition beyond the resources already available? Why would clinicians, researchers, or policy-makers choose MERIT over well-established alternatives?

 Q17. Can users download: a) raw isolate-level data; b) aggregated resistance percentages; c) association rule outputs; or d) network graph data? Is there an API for programmatic access to enable integration with other tools? Can external researchers contribute new surveillance data to expand geographic or temporal coverage?

 Q18. Since it ends in 2022, how will the dashboard be kept up to date with newer surveillance data? Is there a sustainable pipeline for continuous data integration, or is this a static snapshot? What is the long-term maintenance plan for MERIT?

Is the work clearly and accurately presented and does it cite the current literature?

Yes

If applicable, is the statistical analysis and its interpretation appropriate?

Partly

Are all the source data underlying the results available to ensure full reproducibility?

Partly

Is the study design appropriate and is the work technically sound?

Partly

Are the conclusions drawn adequately supported by the results?

Partly

Are sufficient details of methods and analysis provided to allow replication by others?

Partly

Reviewer Expertise:

AMR, Bioinformatics, CADD, ML

I confirm that I have read this submission and believe that I have an appropriate level of expertise to confirm that it is of an acceptable scientific standard, however I have significant reservations, as outlined above.

# Response

**Published:** 2025-12-17

**Authors:** Suma Mohan

**Affiliations:** Division of Bioinformatics, SASTRA Deemed to be University, Thanjavur, Tamilnadu, India

Thank you very much for reviewing our manuscript entitled "Exploring the evolution of multidrug resistance patterns in ESKAPEE pathogens using association mining: Key to antibiotic stewardship?" and for providing the opportunity to revise it. With this, we enclosed a point-by-point response to the reviewer’s concerns for publication in your esteemed journal. We would like to take this opportunity to express our gratitude to the reviewer for the feedback, helpful comments, and suggestions. We believe that we did our best to remove all the ambiguities and made changes that clearly improved our manuscript, which I have uploaded alongside this document.

 Q1. The manuscript identifies sMDR patterns across species (Figure 6), but the dashboard only allows single-organism queries. Author should clarify: (a) Was Figure 6 created by running separate analyses per organism and manually comparing networks? (b) Why can't users perform this cross-species analysis directly in the dashboard? (c) How do author computationally define "shared" patterns beyond visual overlap? (a) Was Figure 6 created by running separate analyses per organism and manually comparing networks?

**Response:** Yes, Figure 6 was created by running separate association mining analysis for each organism separately and manually comparing networks for the identification of any overlaps.  To our knowledge, the antibiotic panels used and the completeness of testing varied substantially across species, as MIC values for the same antibiotic differ between species. So, we performed association rule mining separately for each species. b) Why can't users perform this cross-species analysis directly in the dashboard?

**Response: **Since the dashboard is constructed using surveillance data from ATLAS and Venatorx datasets, antibiotic susceptibility panels differ substantially across each species, and the amount of data that we got for each sample is substantially varied across each sample. So, when multiple species are pooled, the testing matrix becomes highly sparse and heterogeneous, which impacts support and lift calculations and may yield misleading rules. So, with this limitation, we were not able to perform this association mining for cross-species analysis directly in the dashboard. (c) How do author computationally define "shared" patterns beyond visual overlap?

**Response: **The concept of sMDR that we showed here is “shared rules” across species (i.e., same antecedent to consequent, same resistance labels with similar thresholds to highlight co-resistance patterns observed across multiple pathogens. We have clearly stated this concept in the methodology section. "Rules containing at least two antimicrobials were extracted from the dataset and filtered using quality metrics, including support (≥0.3), lift (≥2), confidence (≥0.80), and Zhang’s metric (>90) to derive the best rules."

 2. With thousands of rules generated per organism (e.g., A. baumannii n=3,132), how author control for false discovery rate?. Are multiple testing corrections applied?

**Response:** Thank you for pointing this out. We acknowledge that the current study does not apply any statistical analysis, and we will include this as a limitation. We did effect-size-based filtering to control false positives. In addition, we set thresholds as high lift, high confidence, in order to reduce spurious associations. In the future version of the MERIT dashboard, it is possible to integrate statistical testing. However, this will depend on what is allowed under our current data-use agreement and the licensing terms of the dataset.

 Q3. Author mentioned support >0.1 due to infrequent resistance. However: (a) What quantitative evidence supports this threshold? (b) Was sensitivity analysis performed? (c) Why do many queries still return "No rules found"? (d) Paper mentions both support ≥0.3 and >0.1 - which was used and why the discrepancy? (a) What quantitative evidence supports this threshold?

**Response: **Thank you for this important question. The support thresholds in our study were not fixed globally but chosen based on the size and sparsity of each organism-specific dataset. Some organisms had a large number of isolates and produced ample rules even at a higher threshold (e.g., support ≥0.3), whereas others had very limited data. For these smaller datasets, a higher support cutoff eliminated almost all potential rules, so we used a lower threshold (support ≥0.1) to retain meaningful associations without over-relaxing the filter.

 (b) Was sensitivity analysis performed?

**Response: **We acknowledge that this approach was pragmatic rather than derived from a single quantitative benchmark, and we did not perform a formal sensitivity analysis across all organisms.

 (c) Why do many queries still return "No rules found"?

**Response:** The variation in support thresholds explains why certain dashboard queries still return “No rules found” -in some subsets, the data density is simply too low for the minimum support requirement, even at the relaxed level, to yield any frequent item sets.

 (d) Paper mentions both support ≥0.3 and >0.1 - which was used and why the discrepancy? **Response: **To avoid confusion, we clarified in the “Association Rule Mining” subsection of the “Methods” section that both thresholds were used, depending on the dataset size. A support cutoff of ≥0.3 was used to identify robust patterns of antimicrobial resistance across years, age groups, and geographical locations. In addition, a lower support threshold (>0.1) was used to generate both ‘Resistant-Resistant’ and ‘Resistant-Susceptible’ patterns

 Q4. The Methods mention that isolates without MIC values were excluded but those with MIC values, yet a missing phenotype were manually assigned. How many of the isolates had complete testing profiles for all the antibiotics tested? How might differential testing across organisms-for example, organism A being tested against 20 antibiotics and organism B tested against 15-affect comparability of association rules? Can incomplete testing matrices lead to biased discovery of co-resistance patterns or spurious sMDR patterns? (a)The Methods mention that isolates without MIC values were excluded but those with MIC values, yet a missing phenotype were manually assigned. How many of the isolates had complete testing profiles for all the antibiotics tested?

**Response:** Thank you for raising this important point. From the full ATLAS/Venatorx extract, approximately 6 lakh isolates entered our pipeline. Of these, 598519 isolates had at least one MIC measurement and were retained, while other isolates lacking MIC values entirely were excluded. For isolates that had MIC values but were missing categorical phenotype labels (R/S/I), we assigned phenotypes using year-matched CLSI breakpoint tables (we will cite the specific yearly versions used in the revised Methods). Only isolates lacking an MIC for a given antibiotic were excluded for that specific antibiotic analysis.

 (b)How might differential testing across organisms-for example, organism A being tested against 20 antibiotics and organism B tested against 15-affect comparability of association rules?

**Response:** We acknowledge that organisms were not uniformly tested across the same antibiotic panels-for example, some species were routinely tested against ~20 agents, whereas others were tested against 12–15. This heterogeneity necessarily affects the comparability of association rules across organisms and cannot be fully harmonised with the available data. To minimise the impact of this differential testing and reduce biased discovery of co-resistance patterns:

1. **Cross-species comparisons (e.g., Figure 6)** were restricted to antibiotics that were commonly tested across the species being compared.
2. **The dashboard reports per-query sample counts** and automatically flags queries that fall below a minimum co-testing threshold.
3. **Rules were only generated from isolate–antibiotic pairs with available MIC data**, preventing imputation of resistance where the drug was never tested.

(c)Can incomplete testing matrices lead to biased discovery of co-resistance patterns or spurious sMDR patterns?

**Response:** Despite these measures, we agree that incomplete testing matrices can still influence the number and strength of detected rules and may potentially bias sMDR patterns. We will include this explicitly as a limitation of the dataset and the analysis framework.

 Q5. Many surveillance programs utilize risk-based testing strategies in which carbapenems are tested only when resistance to first-line agents is identified. Might this conditional testing generate spurious associations, where carbapenem resistance seems associated with other resistances just because both are preferentially tested in already-resistant isolates? How do authors separate real biological co-resistance from testing ascertainment bias?

**Response: **Yes, conditional testing can indeed create spurious associations. We acknowledge that carbapenems are often tested only when first-line resistance is detected, which may inflate their apparent co-resistance patterns. Complete correction for conditional testing bias is not possible with the current ATLAS/Pfizer dataset. In future work, this could be addressed by using datasets with uniform antibiotic testing or by employing statistical frameworks that explicitly model the testing decision process. However, to avoid this, in our analysis, we attempted to reduce this risk using stringent effect-size thresholds (lift, confidence, and Zhang metrics), which help filter out rules that arise purely from sparse or artificially enriched testing patterns. We also cross-checked several high-strength rules against published literature to confirm that they reflect known biological co-resistance mechanisms. At the same time, we identified some patterns that are novel and still require external validation.

**Reproducibility & Transparency:**

 Q6. Can other researchers reproduce author’s association rule mining results? Is the complete analysis code openly available, such as a GitHub repository? Are the specific data preprocessing steps, MIC breakpoint assignments, and phenotype categorization rules for the Pfizer-ATLAS and Venatorx-GEARS datasets fully documented to enable independent replication?

**Response:** Thank you for highlighting the importance of reproducibility. The raw MIC dataset used in this work was accessed through the Vivli AMR Challenge (Pfizer and ATLAS datasets). Under the Vivli data-use agreement, we are not permitted to publicly redistribute the original isolate-level data, and therefore, the full dataset cannot be uploaded to GitHub or shared directly. To ensure reproducibility while complying with the data-sharing policy, we have made the complete analysis pipeline publicly available. The complete association mining scripts and dashboard generation scripts have been deposited in GitHub. In the future, we plan to upgrade MERIT along with a detailed README describing step-by-step instructions. The MIC breakpoints used for phenotype categorization strictly follow the CLSI/EUCAST guidelines, and citations to the exact guideline documents have been included in the Methods section. A data preprocessing workflow chart has also been added in the Materials and Methods for clarity.

 Q7. For the MIC breakpoint interpretation, which guidelines were followed? For example, CLSI, EUCAST? Were year-specific versions utilized, given data spans 2004-2022, a period when breakpoints changed for key antibiotics? How does the evolution of these guidelines affect temporal trend analysis? (a) For the MIC breakpoint interpretation, which guidelines were followed? For example, CLSI, EUCAST? Were year-specific versions utilized, given data spans 2004-2022, a period when breakpoints changed for key antibiotics?

**Response:** Thank you for raising this important point. For phenotype assignment, we matched MIC values to the year-specific CLSI tables to avoid misclassification. The specific CLSI yearly versions used will be included as citations in the Methodology part.

**Stratified rule mining** – In the dashboard, rule extraction can be performed *year-wise*, *country-wise*, or on the *entire dataset*. For year-wise mining, the breakpoint changes do not affect the internal consistency of any given year. (b) How does the evolution of these guidelines affect temporal trend analysis?

**Response: **Yes, changes in CLSI breakpoints over the years can affect temporal trend interpretation. We therefore performed year-to-year trends that may partially reflect breakpoint changes and interpret temporal comparisons cautiously by treating them descriptively rather than as strict statistical comparisons. Q8. How many the isolates contributed to author’s result for Switzerland+A. baumannii+Tigecycline+Year 2022? The interface says, "No rules extracted". Is it because of lack of data or too strict thresholds of author’s parameters? What sample size would author recommend in general for a reliable production of rules for an organism-country-year-antibiotic combination? Should the dashboard show some kind of data sufficiency warning or sample size indication before a user starts to invest time into a query?

 (a) How many the isolates contributed to author’s result for Switzerland+A. baumannii+Tigecycline+Year 2022? The interface says, "No rules extracted". Is it because of lack of data or too strict thresholds of author’s parameters?

**Response:** Thank you - in some country/year/antibiotic combinations (for example, Switzerland + *A. baumannii* + Tigecycline + 2022), the dashboard returned ‘No rules extracted’ because the subset contained too few isolates to meet the mining thresholds. For that specific query, we had 9 isolates with Tigecycline MICs; given our support threshold, this count is insufficient to produce frequent itemsets. Both limited data and the deliberately stringent thresholds (support/lift/confidence/Zhang) can produce empty results. 

 (b) What sample size would author recommend in general for a reliable production of rules for an organism-country-year-antibiotic combination?

**Response:** Regarding sample size: a fixed number cannot be recommended because it depends on the prevalence of resistance and the chosen support value. However, as a general guideline, very small subsets (e.g., <30 isolates) often produce no itemsets even if rules exist biologically.

 (c) Should the dashboard show some kind of data sufficiency warning or sample size indication before a user starts to invest time into a query?

**Response:** For transparency, we explicitly mentioned in the “Methods” section that the dashboard reports ‘No rules extracted’ whenever the data supporting a query is insufficient or fails to meet the thresholds. Users should interpret such outputs as a reflection of real surveillance sparsity, not a computational error. Q9 Have author validated identified sMDR patterns against known resistance mechanisms or genetic data? More specifically, do the co-resistance patterns highlighted in Figure 6 (levofloxacin- meropenem- ampicillin/sulbactam-piperacillin/ tazobactam across A. baumannii, K. pneumoniae, and E. faecium) correspond to known resistance genes, plasmids, integrons, or mobile genetic elements?

 How would author differentiate true sMDR from horizontal gene transfer versus independent evolution to a similar resistance phenotype?

**Response:** No, we didn't validate the identified sMDR patterns against known resistance mechanisms or genetic data. Since the number of rules generated for some of the sMDR is greater, it will be difficult to validate each identified sMDR pattern. Since our association mining was performed solely on MIC-based phenotype data, genomic data were not incorporated. Therefore, while literature supports many of the observed co-resistance patterns, it is not possible to distinguish whether shared resistance arises from horizontal gene transfer or independent evolution using this dataset.

 Q10. What is the negative predictive value and the confidence interval for the "Resistant to Susceptible" (R→S) rules discussed in the Discussion, such as "if resistant to Colistin+Ampicillin, then susceptible to Meropenem"? How reliable are these rules in guiding clinical antibiotic selection? Should the dashboard show prediction intervals or uncertainty estimates to inform appropriate clinical use?

**Response: **Confidence intervals are 80 percent (both S to R, R to S). We did not use any negative prediction value. Furthermore, wet lab experiments are required to confirm the findings of this study. These are exploratory, hypothesis-generating rules, and the dashboard shows association strength metrics (support, confidence, lift), which indicate reliability. It requires additional statistical modeling or validation (wet-lab confirmation) for ideal clinical guidance

 Q11. The interface has "Select Year: 2022" for discrete year selection. Are users able to query how the association rules themselves have varied over time? For instance, can users visualize that "levofloxacin→meropenem had lift=1.5 in 2004 but lift=2.8 in 2022"? Is there an ability in place to track the emergence, strengthening, or weakening of specific resistance associations across the period of surveillance?

**Response:** Thank you for the suggestion. At present, the dashboard displays association rules separately for each year. We did not incorporate longitudinal tracking of individual rules because the antibiotic testing panels vary across the surveillance period, and several antibiotics are not consistently available from year to year. As a result, direct year-to-year comparisons could be unreliable. For this reason, the interface focuses on providing clear, year-specific rule exploration rather than analyzing temporal trends.

**Dashboard Usability & Design:**

 Q12. The network graphs have different numbers of rules: E. faecium with n=1,752, S. aureus with n=5,158, and K. pneumoniae with n=4,857. How can these complex networks be navigated within the dashboard? Are there active filtering options, such as "show only rules with lift >3" or "hide nodes with <5 connections", a search function, or even hierarchical layout to improve interpretability?

**Response: **Thank you for the question. The network graphs in the dashboard display all extracted rules for a selected organism. To aid interpretability, the dashboard allows users to restrict the view to subsets of antibiotics or focus on a specific country, year, or age group to reduce network complexity. Users can also able to filter rules by key parameters such as support, confidence, lift, and Zhang metrics before graph generation. However, advanced options such as hierarchical layouts, node hiding based on connectivity, or keyword search within networks are not currently implemented. 

 Q13. In the case of the dashboard showing "No rules found, try with new parameters," there is no actionable guidance for the user. Please provide specific suggestions regarding: a) which parameters to change (support versus lift versus minimum support); b) which direction to go; and c) whether the problem is not enough data or too restrictive thresholds. Consider adding context-sensitive help or suggestion algorithms for parameters.

**Response:** Thank you for this suggestion. In the current version of MERIT, when a query returns ‘No rules found,’ the dashboard does not provide detailed guidance on adjusting parameters. We acknowledge this limitation. In future upgrades, we plan to implement context-sensitive guidance to assist users, including suggestions on which parameters to adjust (support, lift, confidence thresholds), the direction in which to change them, and indications of whether empty results arise from insufficient data or overly restrictive thresholds. This will improve usability and help users explore sparse or complex datasets more effectively.

 Q14. All tabs appear with the same styling-white background and blue text-regardless of which page is active. In the absence of any visual indicators like highlighting, a different color for the background, bolding of text, or an 'active' state indicator, how is a user supposed to maintain spatial awareness regarding their current location while traversing between the six tabs?

**Response:** Thank you for pointing this out. We acknowledge that the current dashboard does not visually highlight the active tab, which may affect user orientation. This is a known usability limitation. In future updates, we plan to enhance the interface with visual indicators such as active tab highlighting or different styling to improve spatial awareness while navigating between tabs

 Q15. The USA contributed 17% of the isolates while many countries contributed < 1%. How does this geographic imbalance affect the reliability of country-specific association rules? Should results be presented with sample-size-weighted confidence or should the dashboard display warnings when country-specific queries fall below recommended thresholds? (a) The USA contributed 17% of the isolates while many countries contributed < 1%. How does this geographic imbalance affect the reliability of country-specific association rules?

**Response:** Thank you for the comment. We acknowledge that geographic imbalance in the dataset (e.g., USA contributing 17% of isolates while many countries contribute <1%) can affect the reliability of country-specific association rules. To address this, the dashboard allows users to perform country-specific queries for both trend analysis and association rule mining.

 (b)Should results be presented with sample-size-weighted confidence or should the dashboard display warnings when country-specific queries fall below recommended thresholds?

**Response:** While the current version does not implement sample-size-weighted confidence or warnings for queries with very few isolates, we recognize this as a valuable enhancement and plan to incorporate such features in the upgraded dashboard.

**Context & Broader Impact**

 Q16. How does MERIT compare functionally and analytically to other established AMR surveillance dashboards and tools, for example, the Pfizer ATLAS online portal, ECDC EARS-Net, Resistance Map, WHO GLASS? What is MERIT's unique value proposition beyond the resources already available? Why would clinicians, researchers, or policy-makers choose MERIT over well-established alternatives?

**Response:** Thank you for this question. MERIT complements existing AMR surveillance dashboards such as Pfizer ATLAS, ECDC EARS-Net, Resistance Map, and WHO GLASS by focusing on **association rule mining and network visualization of co-resistance patterns** at the isolate level. While other platforms primarily provide aggregate resistance percentages, MERIT enables users to explore **co-occurrence and potential multidrug resistance (sMDR) patterns** across antibiotics within specific organisms, countries, years, and age groups.

 Q17. Can users download: a) raw isolate-level data; b) aggregated resistance percentages; c) association rule outputs; or d) network graph data? Is there an API for programmatic access to enable integration with other tools? Can external researchers contribute new surveillance data to expand geographic or temporal coverage? (a) Can users download: a) raw isolate-level data; b) aggregated resistance percentages; c) association rule outputs; or d) network graph data?

**Response:** Thank you for this question. Users cannot download raw isolate-level data due to the Vivli/ATLAS data-use agreement. However, the dashboard allows users to download **trend analysis images, resistance percentages, association rule outputs, and network graph visualizations.**

 (b) Is there an API for programmatic access to enable integration with other tools?

**Response:** Currently, there is no API for programmatic access. We recognize the value of this feature and plan to include it in future upgrades.

 (c)Can external researchers contribute new surveillance data to expand geographic or temporal coverage?

**Response: **Regarding external contributions, since the raw data cannot be shared, other researchers cannot directly add to the underlying dataset. However, they could **apply the same methodology and scripts to their own MIC datasets** to generate local or institution-specific dashboards, which could complement MERIT and expand coverage indirectly.

 Q18. Since it ends in 2022, how will the dashboard be kept up to date with newer surveillance data? Is there a sustainable pipeline for continuous data integration, or is this a static snapshot? What is the long-term maintenance plan for MERIT?

**Response:** At present, MERIT is built using surveillance data available up to 2022, and therefore, the current version functions as a static snapshot. The Vivli data-use agreement does not provide an automated pipeline for obtaining updated Pfizer-ATLAS datasets, so continuous real-time integration is not possible. However, the MERIT framework and scripts are modular, meaning that if future ATLAS datasets or other surveillance datasets become accessible under similar agreements, the dashboard can be rapidly updated by rerunning the processing and rule-mining pipelines. Also, we are planning to upgrade the existing dashboard, which would also support loading local datasets, allowing external researchers to maintain and update their own versions without depending on a centralized data feed.
